# Imaging Flow Cytometric Identification of Chromosomal Defects in Paediatric Acute Lymphoblastic Leukaemia

**DOI:** 10.3390/cells14020114

**Published:** 2025-01-14

**Authors:** Ana P. A. Simpson, Carly E. George, Henry Y. L. Hui, Ravi Doddi, Rishi S. Kotecha, Kathy A. Fuller, Wendy N. Erber

**Affiliations:** 1 School of Biomedical Sciences, The University of Western Australia, Crawley, WA 6009, Australia; 2 Department of Clinical Haematology, Oncology, Blood and Marrow Transplantation, Perth Children’s Hospital, Perth, WA 6009, Australia; 3 PathWest Laboratory Medicine, Nedlands, WA 6009, Australia; 4 Medical School, The University of Western Australia, Crawley, WA 6009, Australia; 5 Curtin Medical School, Curtin University, Bentley, WA 6102, Australia

**Keywords:** acute lymphoblastic leukaemia, hyperdiploid, *ETV6::RUNX1*, imaging flow cytometry, immuno-flowFISH

## Abstract

Acute lymphoblastic leukaemia is the most common childhood malignancy that remains a leading cause of death in childhood. It may be characterised by multiple known recurrent genetic aberrations that inform prognosis, the most common being hyperdiploidy and t(12;21) *ETV6::RUNX1*. We aimed to assess the applicability of a new imaging flow cytometry methodology that incorporates cell morphology, immunophenotype, and fluorescence in situ hybridisation (FISH) to identify aneuploidy of chromosomes 4 and 21 and the translocation *ETV6::RUNX1*. We evaluated this new “immuno-flowFISH” platform on 39 cases of paediatric ALL of B-lineage known to have aneuploidy of chromosomes 4 and 21 and the translocation *ETV6::RUNX1*. After identifying the leukaemic population based on immunophenotype (i.e., expression of CD34, CD10, and CD19 antigens), we assessed for copy numbers of loci for the centromeres of chromosomes 4 and 21 and the *ETV6* and *RUNX1* regions using fluorophore-labelled DNA probes in more than 1000 cells per sample. Trisomy 4 and 21, tetrasomy 21, and translocations of *ETV6::RUNX1,* as well as gains and losses of *ETV6* and *RUNX1,* could all be identified based on FISH spot counts and digital imagery. There was variability in clonal makeup in individual cases, suggesting the presence of sub-clones. Copy number alterations and translocations could be detected even when the cell population comprised less than 1% of cells and included cells with a mature B-cell phenotype, i.e., CD19-positive, lacking CD34 and CD10. In this proof-of-principle study of 39 cases, this sensitive and specific semi-automated high-throughput imaging flow cytometric immuno-flowFISH method has been able to show that alterations in ploidy and *ETV6::RUNX1* could be detected in the 39 cases of paediatric ALL. This imaging flow cytometric FISH method has potential applications for diagnosis and monitoring disease and marrow regeneration (i.e., distinguishing residual ALL from regenerating haematogones) following chemotherapy.

## 1. Introduction

Leukaemia is the most common malignancy in children, accounting for one-third of all paediatric cancers [1]. The global incidence is 48.4 per million person-years in children up to the age of 14 years. Acute lymphoblastic leukaemia (ALL) is the most common subtype, making up 75–80% of cases, with 85% of these being of B-cell lineage. In general, the prognosis for B-ALL is better than that of T-cell origin, with a 5-year overall survival rate of over 90% [2]. Immunophenotyping to determine lineage and chromosome analysis are both essential in the workup at initial diagnosis [3]. These are generally performed as independent tests, the former by multi-parametric flow cytometry and the latter by G-band karyotyping or fluorescence in situ hybridisation (FISH).

Assessing chromosomal alterations is highly informative in delineating likely prognosis with hyperdiploidy and t(12;21)(p13;q22) *ETV6::RUNX1* being regarded as good prognosis, and others (e.g., involvement of 11q23 *KMT2A*) as inferior. High hyperdiploidy, defined as the presence of more than 51 chromosomes, occurs in 20–30% of cases [4,5,6]. It is characterised by recurrent patterns of non-random trisomies, particularly of chromosomes 4, 10, 14, 17, and 21. Tetrasomies may also occur and most commonly affect chromosome 21, almost universally in the setting of hyperdiploidy [6]. The exact pathogenetic mechanisms of hyperdiploidy are not fully understood, but evidence suggests that it originates during a single aberrant mitosis early in leukaemogenesis [7,8]. The translocation t(12;21)(p13;q22), which gives rise to the fusion of *ETV6* and *RUNX1* genes, is detected in up to 25% of cases of paediatric B-lineage ALL [9,10,11]. The *ETV6::RUNX1* fusion is an initiating event, but it is insufficient on its own for leukaemogenesis [12,13,14,15]. While patients with the *ETV6::RUNX1* translocation serve as a broad category of patients, there are subtypes with secondary lesions that lead to pre-B cell transformation and which may affect therapeutic response [16]. Late relapses occur in up to 20% of t(12;21) *ETV6::RUNX1* patients due to secondary genetic lesions, making this a more clinically heterogeneous disease than initially thought [16,17,18,19]. The secondary alterations include the loss of the normal *ETV6* homologue (62%), gain of *RUNX1* (23%), and duplication of the derivative chromosome 21 (10%) [19].

Cytogenetic analysis for these abnormalities is performed by karyotyping and FISH analyses [2,3,4,5]. For high hyperdiploidy, an increase in FISH spot numbers for chromosomes 4, 10, 14, 17, and 21 is expected [4]. However, for *ETV6::RUNX1,* the fusion is cryptic by standard cytogenetic analysis using G-banding techniques due to similar GC contents of *ETV6* and *RUNX1*. The expected FISH signal pattern using dual fusion probes consists of two fusion signals (*ETV6::RUNX1*) on the der(12) and der(21), and one *ETV6* and one *RUNX1* signal for the uninvolved chromosomes 12 and 21. However, since only a low number of cell nuclei are analysed (commonly 200), FISH is not always sufficiently sensitive to identify more complex translocations or secondary *ETV6* and *RUNX1* aberrations.

Recently, a new methodology has been described that uses high-throughput flow cytometry to perform chromosomal analysis of haematological malignancies [20,21,22,23,24]. This incorporates cell immunophenotyping with FISH probing to directly interrogate specific cell populations for genetic aberrations. The specificity of these analyses has been enhanced by the inclusion of monoclonal antibodies to ensure that the chromosomal signals are only assessed in the specific cells of interest (e.g., CD38/CD138 for plasma cells; CD5/CD19 for chronic lymphocytic leukaemia). They have been reported to show that changes in ploidy (e.g., monosomy; trisomy), chromosomal translocations, and loss of genomic regions can all be detected in cells of interest. This has been reported for multiple myeloma with hyperdiploidy, *IGH* translocations, gains of 1q and deletions of 17p, and for chronic lymphocytic leukaemia, trisomy 12, as well as deletions of 17p [20,21,22,23,24,25]. This single-cell integrated immunophenotype–chromosomal analytical method, which captures digital images of every cell analysed, has demonstrated sensitivity of 1 abnormal cell in 100,000 [22]. This strategy has yet to be assessed in ALL.

Here, we report the development and application of this new imaging flow cytometric FISH method to paediatric ALL. Our aim was to assess the capability of this novel approach to identify numerical abnormalities of chromosomes 4 and 21, the initiating *ETV6::RUNX1* translocation, as well as secondary gains and losses of *ETV6* and *RUNX1* in paediatric B-ALL at diagnosis. The immunophenotypic strategy included CD34, CD10, and CD19 antigen detection to ensure that the leukaemic cell population in the sample was being analysed, and that FISH probes were relevant to the chromosome or locus.

## 2. Materials and Methods

### 2.1. Sample Preparation

Bone marrow aspirate samples from 39 paediatric patients diagnosed with B-lineage ALL were analysed. The cases were selected based on the leukaemic cells expressing CD34, CD10, and CD19 antigens on flow cytometry and cytogenetic testing having demonstrated hyperdiploidy with trisomy 4 and/or 21 (ALL-HH cases; n = 23) or *ETV6::RUNX1* fusion (ALL-TT cases; n = 16). All samples had been cryopreserved and were obtained from the Perth Children’s Hospital Department of Haematology, Oncology and Bone Marrow Transplantation Biobank. The patients were aged from 1.4 to 13.2 years with a male: female ratio of 1:1.3. Experiments were conducted under research ethics approved by the Children and Adolescent Health Services Human Research Ethics Committee (HREC RGS-656) and the University of Western Australia Human Research Ethics Committee (HREC RA/4/1/9098). The samples had been cryopreserved at the collection and recovered at 37° C with 1000 units of DNase (11284932001, Roche, Basel, Switzerland; 10104159001, Roche) prior to analysis. One to five million live cells (identified by negative trypan blue staining) were used for immuno-flowFISH processing. Samples were stained with the Fixable Viability Dye efluor 780 (FVD-efluor780) (65-0865-14, Thermo Fisher Scientific, Waltham, MA, USA) in accordance with the manufacturer’s recommendations.

### 2.2. Immuno-fFISH Protocol

The immuno-flowFISH protocol was performed as per Hui et al. [21]. In brief, thawed cells were incubated with fluorophore-conjugated monoclonal antibodies for 30 min on ice at the manufacturer’s recommended concentration. The antibodies used were to identify the leukaemia cells of B-cell lineage (i.e., CD10-BV605 (562978, BD Biosciences, Franklin Lakes, New Jersey, USA), CD19-Alexa Fluor 647 (302222, Biolegend, San Diego, CA, USA), and CD34-BV421 (562527, BD Biosciences), with CD3-BV510 (740202, BD Biosciences) included as a constitutional control. An amount of 200 µL of 1 mM of bis-sulfosuccinimidyl suberate (BS3) (S5799, Sigma-Aldrich, St. Louis, MO, USA) was added and incubated for 30 min on ice to cross-link the antibodies to their antigen targets. An amount of 1 mL of 100 mM Tris-HCl pH 7.4/150 mM NaCl was slowly added to BS3-suspended cells and incubated on ice for 20 min. Cells were then fixed with 250 µL of 4% formaldehyde and 0.1% Tween 20 for 10 min at room temperature.

For FISH probe hybridisation, cellular DNA was first denatured by resuspending pelleted cells in 100 µL of 0.5 M HCl (pH 7.0) for 20 min at room temperature. An amount of 3ml of ice-cold Phosphate Buffered Saline (PBS) was then added to the cell suspension, and cells were pelleted at 600× *g* for 10 min. The cells were resuspended in 2% *v*/*v* Fetal Calf Serum in PBS, and 150 µL of 0.1% Igepal in 2× saline sodium citrate buffer (SSC) was added and transferred into a PCR tube. The cells were pelleted, and the supernatant was removed and resuspended with DNA FISH probes. The FISH probes used targeted regions near the centromere of chromosomes 4 (CEP4-Spectrum Green or CEP4-Spectrum Orange, Abbott Molecular, Chicago, IL, USA); CON4-Fluorescein, Empire Genomics, Buffalo, New York, USA), 21 (CON21-5ROX, Empire Genomics), and *ETV6*-Fluorescein and *RUNX1*-TAMRA (Empire Genomics) loci. Cells were probed under DNA denaturing conditions (73 °C for 5 min), followed by probe hybridisation for 20–24 h at 37 °C. After washing, the cells were stained with 0.1% SYTOX^TM^ AAdvanced DNA stain (S10274, Thermo Fisher Scientific, Waltham) or SYTO^TM^ Orange 85 (S11366, Thermo Fisher Scientific, Waltham) nucleic acid stain for 20 min at room temperature.

### 2.3. Imaging Flow Cytometry

Cells were acquired with the Cytek^®^ Amnis^®^ ImageStream^®^X MkII (ISX Mk II) (Cytek Biosciences, Fremont, CA, USA) using INSPIRE version 4.1 acquisition software (Cytek Biosciences). The excitation laser powers used were 100 mW (405 nm), 100 mW (488 nm), 200 mW (561 nM), 120mW (642 nM), and 10 mW (785 nM) for all samples. For panels that included the CON21-5ROX probe, a 300 mW (592 nm) laser was used in place of the 200 mW (561 nM) laser. All images were captured using the 60× objective and extended depth of field imaging. A minimum threshold of at least 3000 cells was set for data acquisition.

### 2.4. Data Analysis

**Figure 1:**
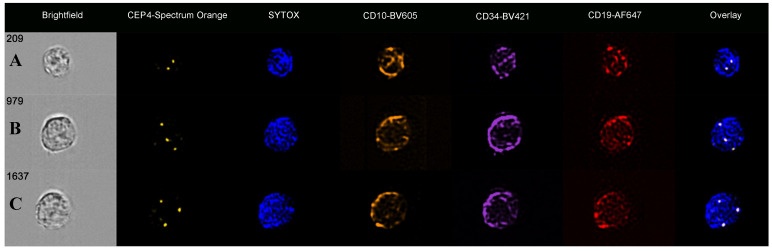
Immuno-flowFISH galleries of CD34/CD10/CD19-positive ALL cells for CEP4-Spectrum Orange FISH probe. (**A**) shows a cell with two FISH signals in keeping with diploid copies of chromosome 4. Cells (**B**,**C**) show three CEP4 FISH signals indicative of trisomy 4. The “Overlay” images show the FISH probe signals overlying the counterstained nuclei.

Images were analysed using IDEAS^®^ v6.2 software (Cytek Biosciences, Fremont, CA, USA) with a modified version of the published immuno-flowFISH protocol for FISH spot counting in phenotypically identified cells [21]. Analysable cells were defined as in-focus, singular, live, and non-dividing with successful probe hybridisation and spot count enumeration per FISH probe signal, as previously described [22]. For the ALL-TT cohort for *ETV6::RUNX1* analysis, the bright detail similarity (BDS) feature was applied to measure the co-localisation of *ETV6* and *RUNX1* spot signals. CD3-positive T cells were used as the internal control for spot counts overlying the SYTOX or SYTO counterstained nuclei for all probes. The BDS was compared between the CD3 control and ALL cells with an increased score indicating overlapping *ETV6::RUNX1* signals and a fusion (Figure 1). Mean comparisons of spot counts and fusions between populations were analysed with a two-tailed unpaired *t*-test. All statistical tests were carried out using GraphPad Prism 8.3.1 (Boston, MA, USA).

## 3. Results

Between 509 and 38,587 cells were acquired for the 39 samples studied with leukaemic cells identified by expression of CD34, CD10, and CD19 antigens (Table 1). FISH signals could be identified for all probes overlying the SYTOX AADvanced or SYTO Orange 85 counterstained nuclei. Analysis of the CD3-positive T lymphocytes gave mean spot counts of 1.6 for CON4 and 1.8 for CON21 (based on 1460 cells) and 2.0 and 1.9 for *ETV6* and *RUNX1,* respectively (from 6187 cells). Where the mean number of FISH spots in the T cells was less than 2.0, this was due to coincident overlapping signals due to alignment with the camera and detection on the digital image galleries. The chance overlap rate for *ETV6* and *RUNX1* FISH signals with dual probe assessment in the CD3-positive cells was calculated as 5.48% ± 0.01% (standard deviation) of cells (see Supplementary Table S1).

**Table 1:** Details of the 23 hyperdiploid cases analysed for CEP4 FISH probe.

Study ID	Age/Gender	% CD34/CD10/CD19 Positive Cells	CEP4 FISH Spot Count (Mean)	Immuno-flowFISH Data Interpretation
ALL-HH-001	2.5/F	97	3.55	Trisomy 4
ALL-HH-002	3/F	90	2.25	Trisomy 4
ALL-HH-003	9.2/F	89	2.41	Trisomy 4
ALL-HH-004	5/F	80	2.11	Trisomy 4
ALL-HH-007	5.6/F	58	3.37	Trisomy 4
ALL-HH-008	3.5/F	90	2.65	Trisomy 4
ALL-HH-009	10.2/M	96	2.80	Trisomy 4
ALL-HH-010	11.2/F	87	2.40	Trisomy 4
ALL-HH-011	2/M	83	2.74	Trisomy 4
ALL-HH-012	3.3/M	84	2.37	Trisomy 4
ALL-HH-013	3.1/F	75	2.46	Trisomy 4
ALL-HH-014	7.2/F	96	2.57	Trisomy 4
ALL-HH-015	5.2/F	83	2.18	Trisomy 4 # *
ALL-HH-016	4.2/M	91	2.33	Trisomy 4
ALL-HH-017	2.7/M	85	2.50	Trisomy 4
ALL-HH-021	1.4/M	75	2.38	Trisomy 4
ALL-HH-022	3.4/F	87	3.08	Trisomy 4
ALL-HH-023	2.3/F	88	2.48	Trisomy 4
ALL-HH-024	3.2/M	92	2.10	Trisomy 4
ALL-HH-026	4.1/F	94	3.62	Trisomy 4
ALL-HH-027	1.7/F	99	2.16	Trisomy 4
ALL-HH-029	6.1/M	95	2.36	Trisomy 4
ALL-HH-034	8.7/M	97	2.10	Trisomy 4
Mean		87	2.66	

# Multiple sub-populations identified (Table 2). * Trisomy and tetrasomy 21 detected with CON21.

**Table 2:** Immuno-flowFISH spot count data for CON4 and CON21 in immunophenotypic subpopulations. Mean spot count >2 infers a gain of chromosomal signals.

ALL-HH-015 Immunophenotyped Populations	Size (%)	CON4 FISH Spot Count (Mean)	CON21 FISH Spot Count (Mean)	FISH Patterns
CD34/CD10/CD19-positive	42.0	2.3	3.0	6
CD34-positive; CD10/CD19-negative	9.9	2.1	1.9	4
CD19/CD10-positive; CD34-negative	6.7	2.3	2.8	5
CD19-positive; CD34/CD10-negative	4.2	1.9	1.9	3
CD3-positive (control)	14.3	1.7	1.8	1

### 3.1. Chromosomes 4 and 21

Chromosome 4 was analysed on CD34/CD10/CD19-positive cells of 23 ALL samples known to have trisomy 4 on standard FISH testing (Table 1). FISH probe analysis showed a mean CEP4 spot count of 2.6 (range 2.1–3.6) in the CD34/CD10/CD19-positive cells, with all cases having cells with more than two probe signals overlying the counterstained nuclei (Figure 1). This confirmed the ability of the technology to identify increased chromosome 4 copies, with all cases having three CEP4 FISH signals in keeping with trisomy 4.

**Figure 2:**
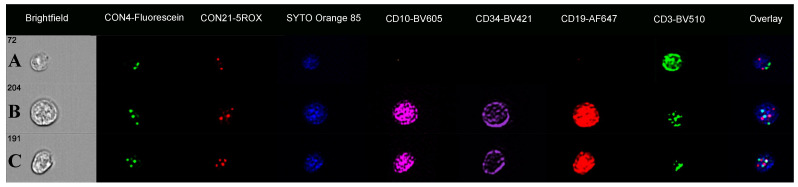
Immuno-flowFISH galleries for CON4 and CON21 dual probe analysis. (**A**) shows a CD3-positive T cell with a normal diploid two FISH signal patterns for both probes. (**B**) shows a CD34/CD10/CD19-positive cell with three FISH spots for both CON4-Fluorescein and CON21-5ROX FISH probes indicating trisomy 4 and 21. Cell (**C**) is a CD34/CD10/CD19-positive cell with trisomy 4 and tetrasomy 21 (three CON4 and four CON21 FISH signals).

We then progressed to determine whether copy number changes could be identified in chromosomes 4 and 21 in the same cell. For this, we selected a hyperdiploid case known to have increased copies of both chromosomes 4 and 21 (ALL-HH-015) and with dual FISH probe analysis with CON4-Fluorescein and CON21-5ROX. Of the 10,000 cells analysed, 14.3% were CD3-positive T cells (14.3% of cells) with mean spot counts of 1.7 for CON4 and 1.8 for CON21 and dual spots on image galleries. There were 42% of cells that expressed CD34, CD10, and CD19 antigens (mean spot counts CON4, 2.3 and CON21, 3.0), which showed three FISH signals for chromosome 4 (CON4) in keeping with trisomy 4, and either 3 or 4 for chromosome 21 (CON21: i.e., trisomy and tetrasomy 21) (Figure 2). Tetrasomy 21 was more common than trisomy 21 and was present in both cells with disomy or trisomy 4 for CON4. 

**Figure 3:**
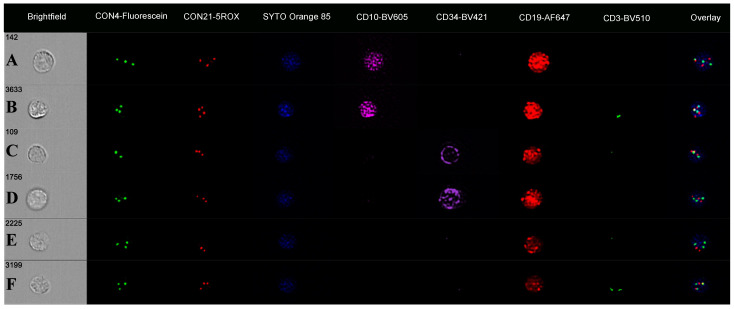
Immuno-flowFISH galleries for CON4-Fluorescein and CON21-5ROX FISH probes in case ALL-HH-015**.** CD10/CD19-positive (CD34 negative) cells in (**A**) with trisomy 4 and 21 and in (**B**) with trisomy 4 and tetrasomy 21. Cells that are CD34/CD19-positive (CD10-negative) with trisomy 21 (**C**) and both trisomy 4 and 21 (**D**). CD19-positive (CD34 and CD10-negative) B cells with trisomy 4 (**E**) and both trisomy 4 and trisomy 21 in (**F**).

A review of the remaining B cells, focusing on those that lacked either CD34 or CD10 antigens, showed a range of chromosome 4 and 21 abnormalities with up to five FISH patterns per cell subset (Table 2; Figure 3). The CD10-negative subset (9.9% of cells; CD34/CD19-positive) had spot counts of 2.1 for CON4 and 1.9 for CON21 and showed trisomy 4, trisomy 21, and diploid FISH spot patterns. In contrast, the CD34-negative cell subset (6.7% cells) had higher spot counts (CON4: 2.3; CON21: 2.8) with trisomy 4 and tetrasomy 21 on imagery. There was also a CD19-positive cell population that was negative for both CD34 and CD10 antigens (4.2% cells). These cells had mean spot counts of 1.9 for CON4 and 1.9 for CON21 (higher than for the CD3 cells), with image galleries showing 26% to have trisomy 4, 18% for trisomy 21, and 6% for both trisomy 4 and 21 (Figure 3).

### 3.2. ETV6::RUNX1 Fusions

**Figure 4:**
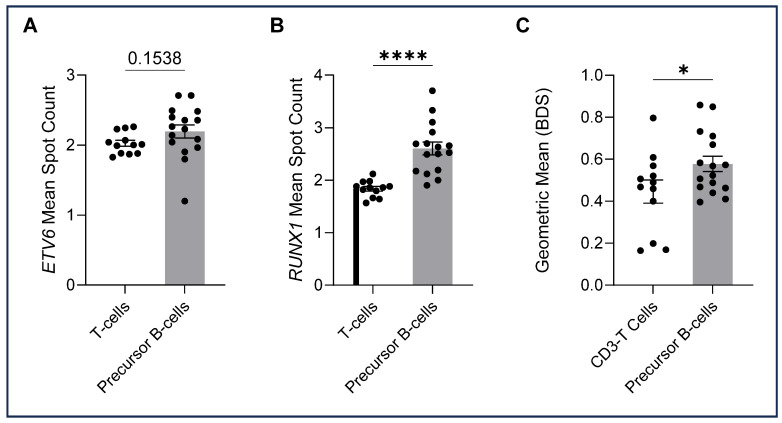
*ETV6* and *RUNX1* spot counts and bright detail similarity (BDS) (means), with each dot representing the mean for cells in each sample. A two-tailed unpaired *t*-test was performed for between-group analysis. (**A**) *ETV6* mean spot counts were not significantly higher in precursor B cells compared with T cells (t(26) = 1.469, *p* = 0.1538). (**B**) *RUNX1* spot counts were statistically higher in precursor B cells than in the CD3-positive T cells (t(26) = 5.215, *p* = 0.000019). (**C**) BDS scores of precursor B cells were higher than those of T cells (t(26) = 2.071, *p* = 0.0484). Significant differences between groups were analysed using a two-tailed unpaired *t*-test. Significant values are denoted as * *p* < 0.05, **** *p* < 0.0001).

The ALL-TT cohort, 16 cases known to have ALL t(12;21), were analysed by immuno-flowFISH for *ETV6::RUNX1* fusions using a dual fusion probe set (i.e., *ETV6*-Fluorescein and *RUNX1*-TAMRA) (Table 3). The CD3-positive T lymphocytes gave mean spot counts of 2.0 for the *ETV6* and 1.8 for the *RUNX1* probe with a low BDS score (mean = 0.45, range = 0.16–0.80) (Figure 4). For the CD34/CD10/CD19-positive cells, we assessed *ETV6* and *RUNX1* FISH spot counts and the BDS score and reviewed the image galleries. A trans-location would be expected to give two fused (2F) signals for the derivative chromosomes and one for each of the uninvolved *ETV6* and *RUNX1* loci, giving a total of 3 FISH spots per cell for each probe. The mean spot count for *ETV6* was 2.2 (range 1.2–2.71) and 2.6 for *RUNX1* (range 1.9–3.7), with a BDS score of 0.58, indicating co-localised chromosomal loci. The *RUNX1* mean spot count and BDS scores were higher than for the CD3-positive T cells (*p* < 0.0001, and *p* = 0.0484), suggesting co-localised chromosomal signals in the precursor B cells (Figure 4C). No significant differences in the *ETV6* mean spot count were observed between CD3-positive T cells and precursor B cells.

**Figure 5:**
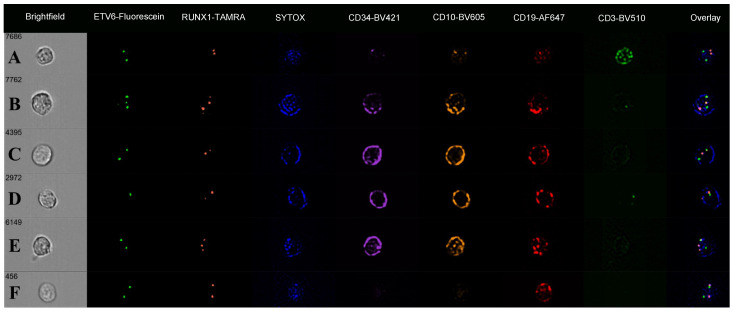
Immuno-flowFISH galleries for *ETV6*-Fluorescein and *RUNX1*-TAMRA FISH probes showing a range of chromosomal defects. The “Overlay” column shows FISH signals overlying the SYTOX nuclear counterstain. Fused *ETV6* and *RUNX1* signals have co-localised Fluorescein and TAMRA, giving a yellow “spot” (as seen in the “Overlay” column for Rows **B**–**F**). Row (**A**) shows a CD3-positive T cell with normal two FISH signal patterns for *ETV6* and *RUNX1*. Rows (**B**–**E**) are CD34/CD10/CD19-positive cells. The cell in (**B**) has three signals for *ETV6* and *RUNX1,* with the “Overlay” image showing these to be two fused *ETV6* and *RUNX1* signals (balanced translocation) and single *ETV6* (green) and RUNX1 (orange) spots. The cell in (**C**) has one overlapping *ETV6* and *RUNX1* (fusion) and single *ETV6* and *RUNX1* spots, indicating an unbalanced translocation. Cell (**D**) has an unbalanced *ETV6*::*RUNX1* translocation with *ETV6* loss, and (**E**) an unbalanced *ETV6*::*RUNX1* translocation with *RUNX1* gain. The cell in (**F**) is CD19-positive (CD34 and CD10 negative) with a non-balanced *ETV6*::*RUNX1* translocation.

The digital image galleries showed overlapping green (*ETV6*) and red (*RUNX1*) FISH signals in the nuclei of precursor B cells, confirming the presence of *ETV6::RUNX1* fusions (Figure 5). The “Overlay” images showed these as yellow signals over the SYTOX counterstain. Some cases showed these as “balanced” with two overlapping (fused) signals and a third signal for the uninvolved *ETV6* and *RUNX1*, giving three spots for both probes (Figure 5B). Other FISH patterns seen included loss of an *ETV6* signal (nine cases; lowest spot count 1.2), *RUNX1* deletion (one case), and *RUNX1* gain (six cases; highest spot count 3.7) consistent with gene amplification. In nine cases, there were both an *ETV6* deletion and *RUNX1* amplification in the same cell (Table 3). In one case (ALL-TT-032), 8% of cells were CD19-positive but lacked detectable CD34 and CD10 signals on immunophenotyping channels. Within this subset of seemingly “mature” B cells, 87% had one or more overlapping *ETV6* and *RUNX1* signals, the pattern of an *ETV6::RUNX1* fusion. They also had other FISH abnormalities, including *ETV6* losses (8% of cells) and gains (14%) and *RUNX1* gains (24%) and losses (8%).

## 4. Discussion

Immuno-flowFISH is a single-cell imaging flow cytometric method that can analyse large numbers of cells and incorporate chromosomal analysis by FISH with immunophenotyping. This simultaneous assessment of phenotype adds specificity to ensure the genomic defect is assessed in cells with the antigenic profile of interest (i.e., abnormal or control T cell). Here, we report the first application of this method to analyse B-lineage ALL to detect aneuploidy (trisomy; tetrasomy), a chromosomal translocation and secondary structural chromosomal gains and losses. In all cases, the primary chromosomal abnormality could be detected based on both quantitative as well as qualitative (imagery) alterations in the FISH signal pattern. This study has, therefore, demonstrated that this novel imaging flow cytometric approach has the capacity to resolve the diverse genetic landscape of ALL with hyperdiploidy with alterations in chromosome 4 and 21 copy number and the primary and secondary chromosomal alterations in cases with *ETV6::RUNX1*.

To assess differences in FISH spot patterns between ALL cells and the CD3-positive control T cells, we utilised a numerical spot count algorithm combined with BDS and visual image review. The presence of chance overlap of FISH signals in constitutional control T cells, which has been previously reported for this method, led to mean spot counts of ≤2 for the CD3-positive cells [21]. We also needed to exclude chance overlapping signals for *ETV6* and *RUNX1* to ensure that true fusions could be identified. In the T cells, there was a chance overlap in 5.48% of cells; to accommodate this and ensure only true fusions were seen in the leukaemia cells, we included the BDS score and visual images. With this combination of quantitative and qualitative analyses, we were confident identifying alterations in FISH signal copy number representing alterations in ploidy, gains, losses and translocations in the ALL cells. Concurrent abnormalities could be identified in single cells, including trisomy and tetrasomies for chromosomes 4 and 21, balanced and unbalanced *ETV6::RUNX1* translocations (one versus two fusions, respectively) together with gains and losses of *ETV6* and *RUNX1*. The secondary gains and losses of *ETV6* and *RUNX1*, detected in 15/16 cases, may go undetected by standard FISH yet may be of prognostic significance [19,26].

Cells that express CD19 but lack CD34 and CD10 antigens are generally considered to be mature B cells. It was of interest that, in one case, there were CD19-positive cells that had the same chromosomal defect as those that co-expressed CD34 and CD10 antigens. By standard flow cytometry, CD19-positive, CD34/CD10-negative cells would not be considered “leukaemic”. The detection of the same genetic defects in these cells as the cells with the precursor B-cell phenotype (CD34 and/or CD10) suggests that this subset is derived from the clonogenic cell [27]. Even though this was only a relatively small population of cells, it is potentially clinically important, especially if flow cytometry is used as a means to assess residual disease. The power of combining the immunophenotypic aspect of immuno-flowFISH with chromosomal detection has highlighted the presence of these cells that may otherwise be regarded as normal. Since this result is supported by another study of flow-sorted CD20-positive and CD34-negative B cells in paediatric ALL, it is unlikely that the lack of detectable CD34 and CD10 was artefactual due to technical reasons [27].

Here, we have shown the capability of immuno-flowFISH imaging flow cytometry to detect *ETV6::RUNX1* gene fusions resulting from the t(12;21) translocation. Fusion analyses have only previously been reported for *IGH* translocations in multiple myeloma, which are generally “intact” with dual fusion signals [24,25]. In ALL *ETV6::RUNX1,* unbalanced translocations with only one fusion signal are a common finding. To ensure their identification, we utilised a novel analytical paradigm that incorporated three FISH-based parameters. Firstly, the number of individual *ETV6* and *RUNX1* spot signals was assessed, followed by the BDS score for *ETV6* and *RUNX1* signals. The final assessment was a review of the visual images of individual cells. Using this three-phase interpretative strategy showed that fused *ETV6::RUNX1* signals were generally associated with loss of the derivative (second fusion) chromosome, as well as gains and losses of *ETV6* and *RUNX1* signals, an observation also made by Aydin et al. [28]. The secondary gains and losses of *ETV6* and *RUNX1* could be identified in 15/16 cases, with the most prevalent change being *RUNX1* gains, recapitulating published data where *RUNX1* is commonly amplified, and *ETV6* deleted [20,29]. In 15 cases, there was genomic diversity with a range of defects of *ETV6* and *RUNX1* in the cells; this is in line with the findings of Anderson et al., who showed intra-clonal genetic diversity with up to eight genetic abnormalities in single cells using a slide-based FISH strategy [27]. Since this genetic variation is dynamic, it may have clinical consequences for ongoing care [28,29].

Although the limit of detection was not assessed in this study, other immuno-flowFISH analyses have shown the capacity to detect one genomically abnormal cell with a neoplastic disease phenotype in 100,000 (i.e., chronic lymphocytic leukaemia and multiple myeloma), which is comparable to the 0.001% limit afforded by PCR-based methods [24,30]. If this can be replicated in ALL, it could provide a novel analytical platform for assessing measurable residual disease. The simultaneous analysis of cell phenotype with chromosomal surveillance would mitigate the impact of clonal evolution on the accuracy of residual disease monitoring and address the distinction between ALL and regenerating haematogones. Antigen loss as a result of therapy can render a formerly informative flow cytometry-based monitoring program ineffective. For example, the use of corticosteroids during induction chemotherapy can lead to the downregulation of leukemic markers such as CD10 [31,32]. Anti-CD19 therapies such as blinatumomab can eliminate CD19-expressing leukaemic cells. This treatment effect could be addressed by using other antibodies (e.g., CD22) to identify cells of the B-lineage that may have leukaemia-associated genetic aberrations.

## 5. Conclusions

In summary, we present a new imaging flow cytometric platform for the genomic analysis of ALL for defects of ploidy and chromosomal translocations, illustrated with aneuploidy of chromosomes 4 and 21 and *ETV6::RUNX1*, in cells identified by their immunophenotype. The data show that this strategy extends the utility of FISH through the analysis of large numbers of phenotypically identified cells and gives added value by identifying sub-clones, including secondary gains and losses of genomic regions. These data can be further expanded to include other informative antibodies or FISH probes for other chromosomal aberrations. This will add value to the detection of secondary changes as well as distinguish regenerating haematogones from residual leukaemia.

## Figures and Tables

**Table 3:** Details of the 16 cases analysed for *ETV6::RUNX1*. Precursor B cells were defined by expressions of CD19, CD34, and/or CD10. (ND = not done).

Study ID	Age/Gender	% CD3 T-Cells	% Precursor B-Cells	Mean *ETV6* Spot Count	Mean *RUNX1* Spot Count	BDS
ALL-TT-002	3.8/F	5	88	2.1	1.9	0.57
ALL-TT-003	3.8/F	7	91	1.9	2.1	0.86
ALL-TT-005	2.6/F	ND	94	12	3.3	0.51
ALL-TT-010	3.4/M	6	90	2.0	2.7	0.41
ALL-TT-016	3.6/F	22	72	2.4	3.1	0.49
ALL-TT-017	4.7/M	4	93	2.4	2.0	0.44
ALL-TT-019	2.5/M	5	90	2.1	2.7	0.52
ALL-TT-023	4.8/F	4	91	2.7	2.7	0.85
ALL-TT-032	4.2/M	12	63	2.1	2.2	0.40
ALL-TT-034	6.8/M	43	51	2.5	2.9	0.46
ALL-TT-035	13.2/F	13	68	2.3	2.6	0.67
ALL-TT-037	2.3/M	ND	47	1.8	2.5	0.71
ALL-TT-038	3.6/M	22	49	2.5	2.5	0.57
ALL-TT-039	5.5/M	ND	39	2.4	3.7	0.73
ALL-TT-040	2.2/F	9	63	2.7	2.5	0.47
ALL-TT-042	11.9/F	ND	81	2.2	2.2	0.58
Mean		13	73	2.2	2.6	0.58

## Data Availability

Data presented in this study will be made available from the Corresponding Author (W.N.E.) upon reasonable request.

## Supplementary Materials

The following supporting information can be downloaded at https://www.mdpi.com/article/10.3390/cells14020114/s1, Supplementary data are provided in Supplementary Table S1.
