# Supplementary material for: Imaging Flow Cytometric Identification of Chromosomal Defects in Paediatric Acute Lymphoblastic Leukaemia
Source: Cells. 2025 Jan 14;14(2):114. doi: 10.3390/cells14020114 (PMC11763943; doi:10.3390/cells14020114)
Supplement: Supplementary file 1 [file cells-14-00114-s001.zip › cells-3397158-supplementary.pdf]

Supplementary Materials

Supplementary Table 1: Mean FISH spot counts for CD3-positive T-cells.

Probe	Total Number CD3-positive cells analysed	Mean spot count
CON4-Fluorescein	1,460	1.6
CON21-5ROX		1.9
ETV6-Fluorescein	6,187	2.0
RUNX1-TAMRA		1.8
